# Optical characterizations of PMMA/metal oxide nanoparticles thin films: bandgap engineering using a novel derived model

**DOI:** 10.1016/j.heliyon.2021.e05952

**Published:** 2021-01-15

**Authors:** Qais M. Al-Bataineh, Ahmad.A. Ahmad, A.M. Alsaad, Ahmad D. Telfah

**Affiliations:** a Department of Physics, Jordan University of Science & Technology, P.O. Box 3030, Irbid, 22110, Jordan; b Leibniz Institut für Analytische Wissenschaften-ISAS-e.V., Bunsen-Kirchhoff-Straße 11, 44139, Dortmund, Germany; c Hamdi Mango Center for Scientific Research (HMCSR), the Jordan University, Amman, 11942, Jordan

**Keywords:** Polymethyl methacrylate (PMMA), Metal oxides nanoparticles (MO NPs), Zinc oxide (ZnO), Copper oxide (CuO), Silicon dioxide (SiO_2_), Titanium dioxide (TiO_2_), Optical properties, Bandgap energy, FTIR, TGA

## Abstract

We synthesize and optically characterize pure PMMA and PMMA incorporated with metal oxides nanoparticles (MO NPs) such as ZnO, CuO, TiO_2_ and SiO_2_ NPs nanocomposite thin films with weight concentration of 10% using dip-coating technique. SEM images of MO NPs show that all NPs have nearly an average size of around 50 nm. The optical parameters such as, optical parameters (*n* and *k*), optoelectronics properties, dispersion, band-gap energy and band structure of as-prepared nanocomposite thin films were determined by analyzing the transmittance and reflectance spectra. Mainly, optical band-gap energy (*E*_g_) and the thickness of thin films are evaluated to a high degree of accuracy by utilizing Q-functional derived using a mathematical model recently published. The Q(*E*) is a functional containing experimental transmission and reflection data and the incident photon energy. The *E*_*g*_ value of un-doped PMMA thin films is found to be 4.273 eV. This value decreases as pre-selected MO NPs are introduced into thin films. These values are in excellent agreement with those determined using Tauc method. The FTIR technique is employed to elucidate the vibrational bands of the nanocomposites and the intermolecular bonding between PMMA matrix and the MOs NPs. Thermal stability is investigated by employing thermogravimetric analysis (TGA) at temperatures up to 400 °C. The obtained TGA thermograms indicate that adding MOs NPs to PMMA yield thin films of better thermal stability. The obtained doped thin films show a great promise for fabricating high-efficient optoelectronic devices.

## 1 Introduction

Inorganic-organic nanocomposites have gained a technological robust in the field of linear optics, nonlinear optics and solar cells owing to their outstanding properties and novel applications [1, 2, 3, 4, 5, 6, 7]. Combining one or more metal oxide nanoparticles (MO NPs) with one or more polymer composite leads to a new class of state-of-art nanocomposites. Particularly, the incorporation of polymers with MO NPs such as ZnO, CuO, ZnS, SiO_2_, TiO_2_ and Al_2_O_3_ has been demonstrated to nanocomposites exhibiting excellent electrical, optical and mechanical properties [8, 9, 10]. Polymer composites developed several striking properties such as easy processing, resistance to deformations and organic functionalities. Moreover, MO NPs are well known to possess attractive properties such as stiffness, transparency, good thermal and chemical stabilities. Nanocomposites synthesized by blending polymers with MO NPs exhibit properties of both components to yield multifunctional materials with new or enhanced properties overtaking each single-component independently [11, 12, 13, 14, 15]. Recently, considerable interest has been focused on nanocomposite thin films due to their physical properties and applications in optical lenses, UV shielding, light emitting diodes (LED), photo-detectors, solar cells, multi-sensors, organic transformation reactions, super capacitors and corrosion protections [15, 16, 17, 18, 19, 20, 21, 22, 23, 24, 25, 26, 27]. Various investigations revealed that polymeric-based PMMA is an appropriate host matrix used to comprehend the effect of incorporation or doping such materials on their linear and nonlinear optical properties [28, 29, 30, 31]. The PMMA host matrix offers appropriate micromolecular exchanges with the rooted molecules through enhancing their photophysical properties [29, 32].

Considerable interest has been devoted on designing novel nanocomposite thin films as they play major roles in fabricating micro-optical and optoelectronic components. Owing to their various optical properties such as high/low refractive index, tailored absorption/emission properties and strong optical nonlinearities, they have been of great worldwide [33, 34, 35, 36]. Nanocomposites derived from organic components usually exhibit high refractive indices. Thus, they can be utilized in light emitting diodes [37], lithography [38] and show screens [39]. Owing to their attractive properties, they act as potential candidates for antireflective coatings [40] and metal oxide semiconductor [41]. The introducing MO NPs fillers to the host materials often leads to transparency-losses resulting from the scattering of nanoparticles agglomerates and hence, increases the refractive index [42, 43]. Fabricating nanocomposite thin films with specific controlled optoelectronic properties via densifying integrated optimal MO NPs fillers with polymeric host material increases their index of refraction. Hence, they become of potential applications for devices using high optical transparencies [44, 45, 46].

The optical, dispersion, chemical properties, as well as, identification of the major vibrational modes of PMMA and PMMA incorporated with ZnO, CuO, SiO_2_ and TiO_2_ NPs nanocomposite thin films are the major themes of this work. Major part of this study is devoted for investigating optical parameters such as transmittance, reflectance, optical constants and other related quantities. We employ a mathematical model that we established to investigate the optical bandgap energy of the doped polymeric thin films. We report a method that can be deliberately employed to calculate the film thickness, optical bandgap energy, optical transition lifetime (τ) and the bonding–antibonding energy difference precisely. To elucidate and identify the major vibrational modes, Fourier Transform Infrared spectroscopy (FTIR) is used. The thermal stability of doped polymeric thin films was investigated using thermogravimetric analysis (TGA) technique.

## 2 Experimental procedure

### 2.1 Synthesis of metal oxides nanostructures (MO NPs)

Hydrothermal preparation method is frequently employed for the synthesis of nanostructures. Even though, nanomaterials grown using hydrothermal method is unstable at high temperatures, it is still superior over other methods owing to its ability to produce nanomaterials with high vapor pressures and minimum loss. The elemental composition of as-grown nanoparticles and nano thin films can be strictly monitored by closely observing liquid phase or multiphase chemical reactions [47].

Hydrothermal process was used in preparing ZnO NPs. 1.5 g of Zinc acetate dehydrates [Zn(CH_3_CO_2_)_2_.2H_2_O] (Solution-1) and 0.5 g of NaOH (Solution-2) were separately added to 20 m*l* of absolute ethanol while magnetically stirred for 30 min. The first solution was placed in water-bath of 70 °C temperature for 3 h. The second solution was then added to the first solution dropwise. The accumulated ZnO NPs were separated from ethanol by centrifugation process. The solution containing the NPs was then placed in a furnace at around 100 °C–110 °C in order to produce the powder form of ZnO NPs [48, 49]. Copper oxide NPs were also prepared using hydrothermal process by water-bathing copper (II) nitrate trihydrate [CuH_2_N_2_O_7_] and hexamethylenetetramine (HMT) [(CH_2_).6N_4_] in the aqueous solution (growth solution with distilled water) at a temperature of 90 °C for 5 h. The solution was then cooled down to room temperature. The CuO NPs were then washed with distilled water [50]. Similarly, SiO_2_ NPs were prepared by hydrothermal process by mixing 66 m*l* of ethanol with 4 m*l* of distilled water and placed in water-bath sonicator for 10 min at room temperature. In addition, one gram of sodium hydroxide (NaOH) is separately mixed with ethanol and magnetically stirred until complete dissolving is achieved. The two solutions were then mixed and 4 m*l* of tetraethyl orthosilicate (SiC_8_H_20_O_4_) was added into the mixture and left in water-bath for 15 min at room temperature. Finally, 4 m*l* of NaOH is added to the solution and left in the water-bath for 30 min more. The solution was then centrifuge for 15 min in order to produce the SiO_2_ [51]. The TiO_2_ NPs were prepared by using sol-gel process in which titanium isopropoxide (IV) (Ti(OCH(CH_3_)_2_)_4_) was diluted in isopropyl alcohol as a starting solution. The solution was then kept under vigorous stirring process for 10 h at room temperature. An addition of alkaline water (pH = 8) has precipitated the production of TiO_2_ NPs. The accumulated TiO_2_ NPs were then separated from solution by centrifugation process. The solution containing the NPs was then placed in a furnace at around 110 °C in order to produce the powder form of TiO_2_ NPs [52].

### 2.2 PMMA incorporated with metal oxide NPs thin films

PMMA and PMMA incorporated with ZnO, CuO, TiO_2_ and SiO_2_NPs NPs composites in the form of thin films with 10% of weight percent of metal oxides nanoparticles were prepared. A stock solution of PMMA in THF was prepared by dissolving 1 g of PMMA in 100 m*l* of THF. Magnetic stirring was performed for about 4 h and a proper amount of metal oxide nanoparticles was added to produce metal oxide networks in PMMA matrices. The mixtures were placed on a stirrer for 2 h and each solution was then transferred to an isolated Petri dish. Thin films are synthesized by immersing the glass substrate in the solution for 2 h. The SEM micrographs are obtained to measure film thickness of around 500 nm. In order to extract thin films from the solvent and organic residue, the films were dried in oven for 15 min at 70 °C.

## 3 SEM micrograph for metal oxides NPs

**Figure 1:**
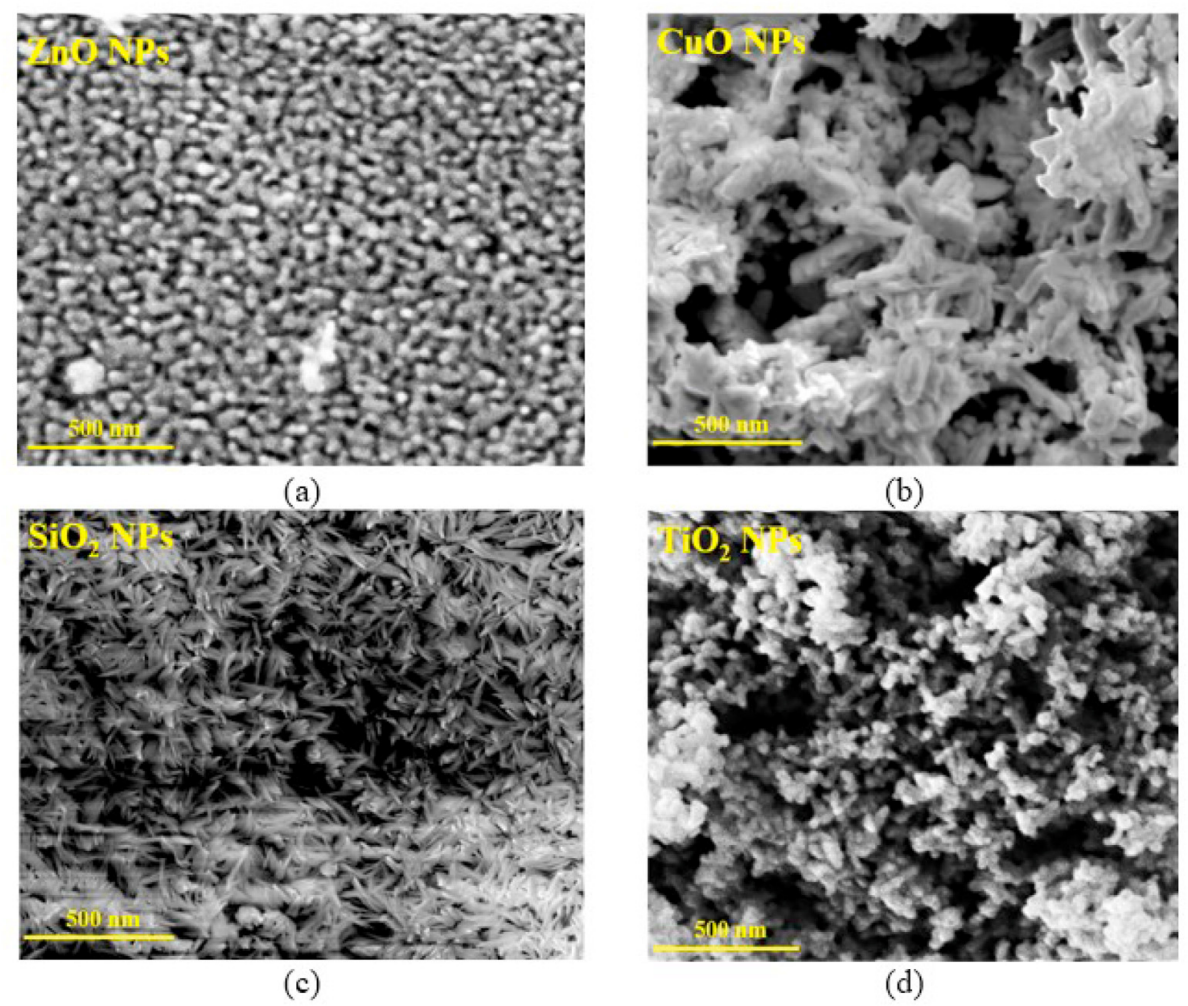
The SEM images of (a) ZnO NPs, (b) CuO NPs, (c) SiO_2_ NPs and (d) TiO_2_ NPs.

The surface morphology of the samples was investigated by using SEM technique with an operating voltage of 10 kV Figure 1 shows the SEM micrographs of ZnO NPs, CuO NPs, TiO_2_ NPs and SiO_2_ NPs, respectively. SEM image shown in Figure 1-a confirms the presence of ZnO NPs due to the obvious spherical shapes with an average size of less than 50 nm and the agglomeration exhibited by the particles. The CuO nanostructures are little oriented towards flower-like shapes as shown in Figure 1-b with leaf size less than 50 nm Figure 1-c shows SEM image of SiO_2_ NPs as a rod shape with rod-length size of less than 50 nm. Finally, Figure 1-d shows the SEM micrograph of scantily observed agglomeration with almost spherical TiO_2_ NPs with a size of less than 50 nm.

## 4 UV-vis spectroscopy

### 4.1 Measurement and interpretation of the optical parameters of MO NPs polymeric nanocomposites thin films

**Figure 2:**
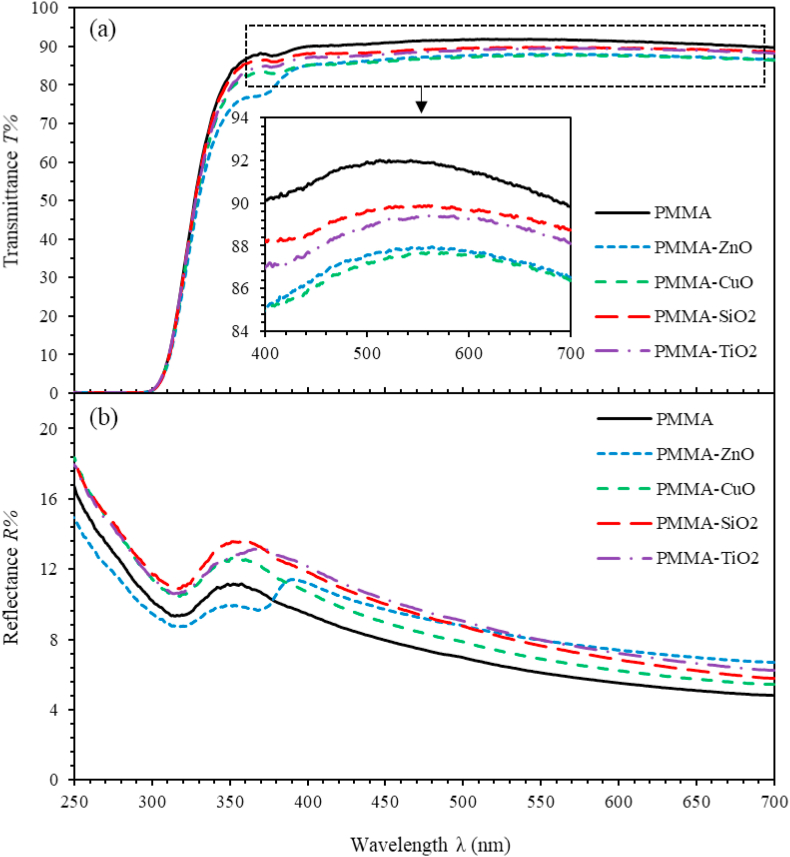
(a) Transmittance spectra and (b) Reflectance spectra of the PMMA and PMMA incorporated with ZnO, CuO, SiO_2_ and TiO_2_ NPs nanocomposites thin films as a function of wavelength.

The transmittance T%(λ) and reflectance R%(λ) spectra of PMMA and PMMA incorporated with ZnO, CuO, TiO_2_ and SiO_2_ NPs nanocomposite thin films were investigated by employing UV-Vis spectrophotometer at room temperature. An integrating sphere is composed of a hollow spherical cavity. The internal part of it sheltered with a prolix white reflective coating. It contains very tiny entrance and exist slits for incident and refractive rays. With such structure, it ensures uniform scattering and can measure diffusing effects with high accuracy. Several theoretical models have utilized experimental transmittance T%(λ) and reflectance R%(λ) data to calculate other key optical parameters of thin films. The optical T%(λ) and R%(λ) of PMMA and PMMA incorporated with ZnO, CuO, TiO_2_ and SiO_2_ NPs nanocomposite thin films are presented in Figure 2. Two interesting spectra regions are of prime importance to analyze and interpret the spectra behavior of T%(λ) and R% . Mainly, in *E* ≥ 3.54 eV (λ≤350nm), PMMA and PMMA incorporated with ZnO, CuO, TiO_2_ and SiO_2_NPs nanocomposite thin films exhibit low transmittance and thus high reflectance as illustrated in Figure 2. Moreover, the absorbance of glass substrate lies in the range ≤ 300 nm. The second, *E*≤ 3.54 eV (λ≥350 nm) in which, as-synthesized thin films exhibit high transmittance and vanishing absorbance. We found that (T%+R%≈1) indicating that our procedure yields thin films of high quality. Nevertheless, as the MO NPs are introduced in PMMA thin films, the absorption edge shifts towards lower energy and as a result, a substantial reduction in the band gap energy is noticed. This red shift may be attributed to the ability of nanocomposite to enhance the charge transfer between MO NPs and PMMA host material that leads to a drastic change in the band structure of the nanocomposite [53]. The transmittance of PMMA thin film is about 92% and decreases as the MO NPs are injected into the PMMA matrix as demonstrated by Figure 2 consistent with other pioneering studies [10, 54, 55, 56, 57]. Figure 2(b) displays the main features of reflectance spectra. The reflectance of PMMA thin films are found to lie in the (4.8%–11.1%) range in the spectral region trailing from 700 nm to 350 nm. It increases as the MO NPs are introduced into the PMMA matrix.

**Figure 3:**
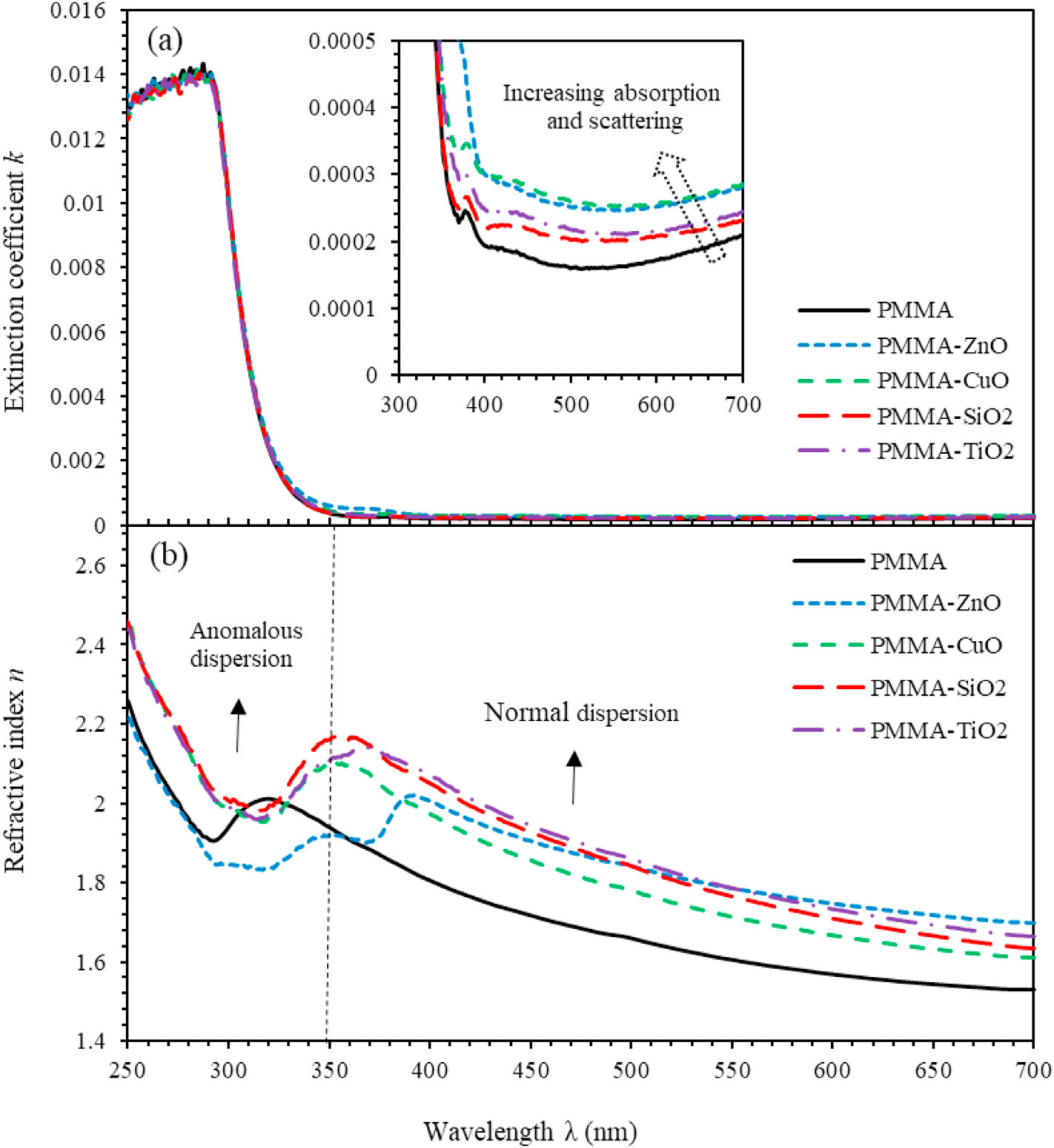
The two key optical parameters, namely, (a) Extinction coefficient, *k* and (b) Refractive index, *n* of the PMMA and PMMA incorporated with ZnO, CuO, SiO_2_ and TiO_2_ NPs nanocomposites thin films versus the wavelength of incident light.

Complex refractive index can be written as the sum of the refractive index *n* and the extinction parameter *k* (N=n+ik) [58, 59]. The parameter *k* is related to absorption coefficient, α by k=αλ/4π whereα=(1/d)ln(1/T) [48]. Figure 3(a) shows that *k* abruptly decreases as λ varies in the range (280 nm–350 nm). Forλ≥350nm, *k* is negligibly small demonstrating hardly any light loss. As MO NPs are introduced into PMMA polymeric matrix, *k* increases indicating high light dissipation due to scattering and absorption by MO NPs centers in agreement with the findings of Mott and Davis [60]. The strong dispensing between the MO NPs and the polymer blend leads to a change in the crystallinity and consequently a change in the band structure and the percentage of absorption [61]. Accurate calculation of n is crucial for the functioning of optical switches and filters [58, 59, 62]. It can be calculated as$\;n=\left(1+R/1-R\right)+\sqrt{\left(4R/{\left(1-R\right)}^{2}\right)-k^{2}}$. Figure 3(b) shows that *n* exhibits a normal dispersion in long λ zones and an anomalous dispersion in short λ regions. Furthermore, for λ<350nm, *n* exhibits an increasing value with the optimum attained as the frequency of the incident photons resonates with plasma frequency of the oscillating electric dipoles. For λ≥350nm, *n* decreases abruptly (normal dispersion). Un-doped PMMA thin films exhibit a wide range of *n* (1.53–1.97). As the MO NPs are implanted and distributed homogenously in PMMA matrices as proven by SEM measurements, *n* increases. This behavior is attributed to the formation of clusters and agglomerations of NPs throughout the matrix [63, 64, 65].

### 4.2 Optoelectronic parameters and dispersion models

The dispersion phenomena of *n* of thin films is a crucial factor for designing highly effective optical devices and to elucidate the related key optical parameters, [66, 67]. These parameters are calculated using Wemple–DiDomenico (WDD) model [68]. In particular, effective single oscillator energy ($E_{0}$) and the dispersion energy ($E_{d}$), zero-frequency refractive index, zero-frequency dielectric constant ($\varepsilon_{0}$) and the optical dipole moments [69] are calculated in this work as described in Eq. (1),

(1) $${\left(n^{2}-1\right)}^{-1}=\frac{E_{0}}{E_{d}}-\frac{hv^{2}}{E_{0}E_{d}}$$

**Figure 4:**
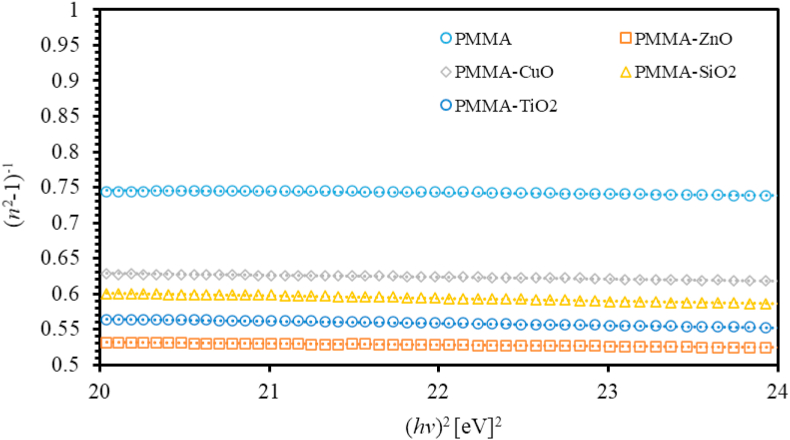
The ${\left(n^{2}-1\right)}^{-1}$ versus $hv^{2}$ of PMMA and PMMA incorporated with ZnO, CuO, SiO_2_ and TiO_2_ NPs nanocomposites thin films.

Therefore, plotting ${\left(n^{2}-1\right)}^{-1}$versus ${\left(hv\right)}^{2}$ yields a linear relationship that can be fitted to calculate the dispersion parameters. Figure 4 shows ${\left(n^{2}-1\right)}^{-1}$ versus ${\left(hv\right)}^{2}$ for PMMA and ZnO, CuO, TiO_2_ and SiO_2_ NPs nanocomposites incorporated with PMMA thin films, respectively. The obtained values of $E_{d}$ and $E_{0}$ of all the thin films are tabulated in Table 1. $E_{0}$ of PMMA, thin film is found to be 19.375 eV that decreases as MO NPs are added into PMMA matrix. This behavior is predictable owing to the formation of stiffer bonds among the components of the PMMA/MO NPs [58]. Furthermore, the dispersion energy $E_{d}$ also decreases as metal oxides nanoparticles are injected into the PMMA matrix. This decrease associated with the increase if the concentration of the nanocomposite is attributed to the increase of porosity of the nanocomposite thin films. The obtained values of $E_{0}$ and $E_{d}$ can be used to determine $\varepsilon_{0}$ and $n_{0}$ as seen in Eq. (2) by rewriting Eq. (1) and usinghv=0,

(2) $$\varepsilon_{0}=n_{0}^{2}=1+\frac{E_{d}}{E_{0}}$$

**Table 1:** Evaluation of key optical parameters of the PMMA and PMMA doped with ZnO, CuO, SiO_2_ and TiO_2_ NPs nanocomposites thin films.

Parameter	PMMA	PMMA-ZnO	PMMA-CuO	PMMA-TiO_2_	PMMA-SiO_2_
Effective single oscillator, $E_{0}$ (eV)	19.375	17.313	16.178	14.212	13.211
Dispersion energy, $E_{d}$ (eV)	24.578	30.400	23.774	22.696	19.408
zero-frequency refractive index, $n_{0}$	1.506	1.660	1.572	1.612	1.571
Zero-frequency dielectric constant, $\varepsilon_{0}$	2.269	2.756	2.469	2.597	2.469
Optical Moments, ${\text{M}}_{-1}$	1.269	1.756	1.470	1.597	1.469
Optical Moments, $M_{-3}*10^{-3}\;\left(eV^{-2}\right)$	3.379	5.858	5.614	7.906	8.417
Density of states, $N_{c}/m^{*}*\;10^{+57}$ ($m^{-3}.Kg^{-1}$)	1.056	1.522	1.639	2.112	2.290
Charge carrier density, $N_{c}*\;10^{+26}$ ($m^{-3}$)	4.233	6.100	6.571	8.464	9.170
High-frequency dielectric constant, $\varepsilon_{\infty }$	2.752	3.477	3.232	3.599	3.562

As presented in Table 1, $\varepsilon_{0}$ and $n_{0}$ are consistent with previously reported values. Their values are found to be 2.269 and 1.506, correspondingly. As introducing MO NPs into PMMA thin films, $\varepsilon_{0}$ and $n_{0}$ are increased considerably with various values. This could be associated with the densification of the material [70]. The optical dipole moments $M_{-1}$and $M_{-3}$ of polymeric thin films are deduced as seen in Eqs. (3) and (4),

(3) $${{\text{E}}_{0}}^{2}=\frac{{\text{M}}_{-1}}{{\text{M}}_{-3}}$$

(4) $${{\text{E}}_{\text{d}}}^{2}=\frac{{{\text{M}}^{3}}_{-1}}{{\text{M}}_{-3}}$$

The obtained values of the optical moments $M_{-1}$and $M_{-3}$ are given in Table 1 [71, 72]. The values of optical moments increase as MO NPs are introduced into PMMA matrix indicating that the ZnO, CuO, TiO_2_ and SiO_2_ NPs nanocomposites incorporated with PMMA thin films are highly polarized and serve as scattering centers [42, 43, 73].

**Figure 5:**
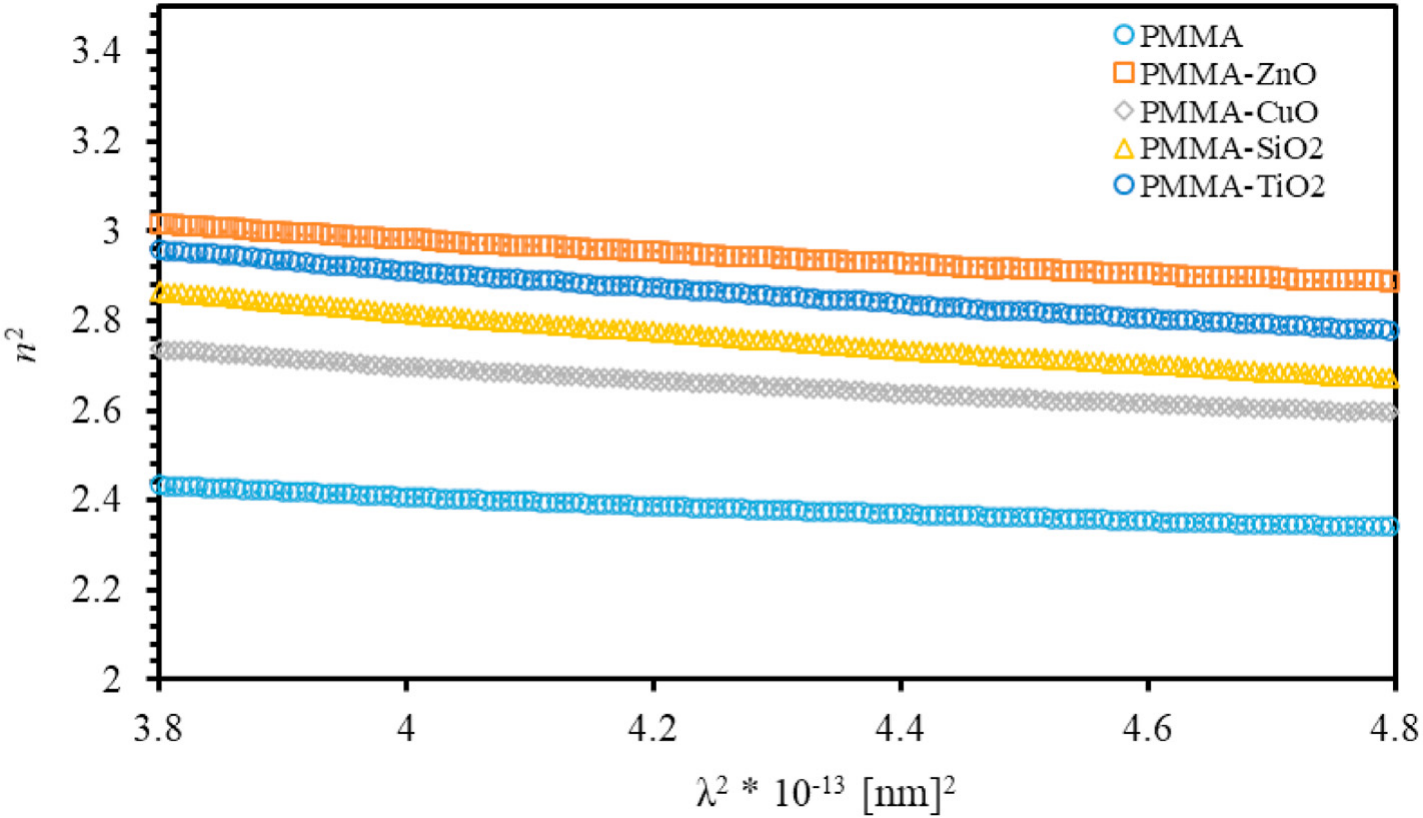
The variation of ($n^{2}=\varepsilon^{\prime }$) with $\lambda^{2}$of PMMA and PMMA incorporated with ZnO, CuO, SiO_2_ and TiO_2_ NPs nanocomposites thin films.

To elucidate the physical meaning of dielectric properties of thin films, we use Spitzer-Fan model to investigate the interplay between n, the density of states ($N/m^{*})$ and the high-frequency dielectric constant $\varepsilon_{\infty }$, the relationship of $n^{2}$ as a function of $\lambda^{2}$ is given in Eq. (5) [74],

(5) $$n^{2}=\varepsilon \prime =\varepsilon_{\infty }-\frac{1}{4\pi^{2}\varepsilon_{0}}\left(\frac{e^{2}}{c^{2}}\right)\left(\frac{N_{c}}{m^{*}}\right)\lambda^{2}$$

where $e=1.6x10^{-19}C$, $c=3x10^{8}\;m/s$, $N_{c}$ is the current carrier density and $m^{*}$ is the effective mass of the carrier. Plotting $n^{2}$ versus $\lambda^{2}$ yields a straight line in the long λ region. Figure 5 displays ($n^{2}=\varepsilon^{\prime }$) versus the square of the photon wavelength ($\lambda^{2}$) of PMMA and MO NPs doped PMMA thin films. The value of $\varepsilon_{\infty }$ of PMMA thin film is 2.752 that increases as MO NPs are added into PMMA thin films as shown in Table 1. The fact that $\varepsilon_{\infty }$ is greater than n confirms that enough polarized charge carriers in PMMA and ZnO, CuO, TiO_2_ and SiO_2_ NPs nanocomposites incorporated in PMMA thin films [69, 75].

### 4.3 Determination and interpretation of optical bandgap

#### 4.3.1 Investigating the bandgap energy using a newly derived mathematical model

For amorphous materials, the main challenge of using the Tauc model is the determination of the transition mode for electrons between valence and conduction bands. In a previous work published recently, the optical bandgap energy and film thickness were evaluated concurrently using transmission spectra of amorphous, crystal semiconductors and dielectric thin films without a pre-knowledge of the transitions executed. For more details of the derivation and interpretation of Eqs. (6), (7), (8), (9), (10), and (11), you may find it useful to refer to our previous works [76, 77],

(6) $$T\left(E\right)=e^{-\;\frac{4\pi dE}{hc}\;\frac{A{\left(E-E_{g}\right)}^{2}}{E^{2}-BE+C}}$$

By using α=1/dln(1/d), but we know that the absorption coefficientα in general case is related directly with the transmission (T) and reflection (R) Eq. (7) [78],

(7) $$\alpha =\frac{1}{d}\ln\left(\frac{{\left(1-R\right)}^{2}}{2T}\right)$$

where, *d* is the thickness of the thin film. Let us consider a new function named as Q-Function given by:

(8) $$Q=\frac{2T}{{\left(1-R\right)}^{2}}$$

Therefore,

(9) $$Q=e^{-\alpha d}$$

Using Eqs. (6), (7), and (9), Q-Function can be written as:

(10) $$Q\left(E\right)=e^{-\;\frac{4\pi dE}{hc}\;\frac{A{\left(E-E_{g}\right)}^{2}}{E^{2}-BE+C}}$$

According to Taylor polynomial series for $e^{x}$, Eq. (10) was rewritten as:

(11) $$Q\left(E\right)=1-\;\frac{4\pi dE}{hc}\;\frac{A{\left(E-E_{g}\right)}^{2}}{E^{2}-BE+C}+\frac{1}{2}{\left[\;\frac{4\pi dE}{hc}\;\frac{A{\left(E-E_{g}\right)}^{2}}{E^{2}-BE+C}\right]}^{2}+\ldots$$

Eqs. (12), (13), (14), and (15) described all constants appear in Eq. (11),

(12) hc=1240eV

(13) $$A=const\frac{2\pi }{3}e^{2}\hbar^{2}{\left|\sigma \prime^{*}|\vec{x}|\sigma^{\prime }\right|}^{2}\gamma$$

(14) $$B=2\left(E_{\sigma \prime^{*}}-E_{\sigma^{\prime }}\right)$$

(15) $$C={\left(E_{\sigma \prime^{*}}-E_{\sigma^{\prime }}\right)}^{2}+\frac{\hbar^{2}\gamma^{2}}{4}={\left(E_{\sigma \prime^{*}}-E_{\sigma^{\prime }}\right)}^{2}+\frac{\hbar^{2}}{4\tau^{2}}$$

Careful examination of Eq. (11) clearly indicates that the variation of Q-function with the energy of incident photon is attributed to the film thickness (*d)*, the optical bandgap energy ($E_{g})$, the bonding-antibonding difference in energy states ($E_{\sigma \prime^{*}}-E_{\sigma^{\prime }}$), the lifetime (τ) and the quantity (*A*) given in terms of the position matrix and τ [79].

**Figure 6:**
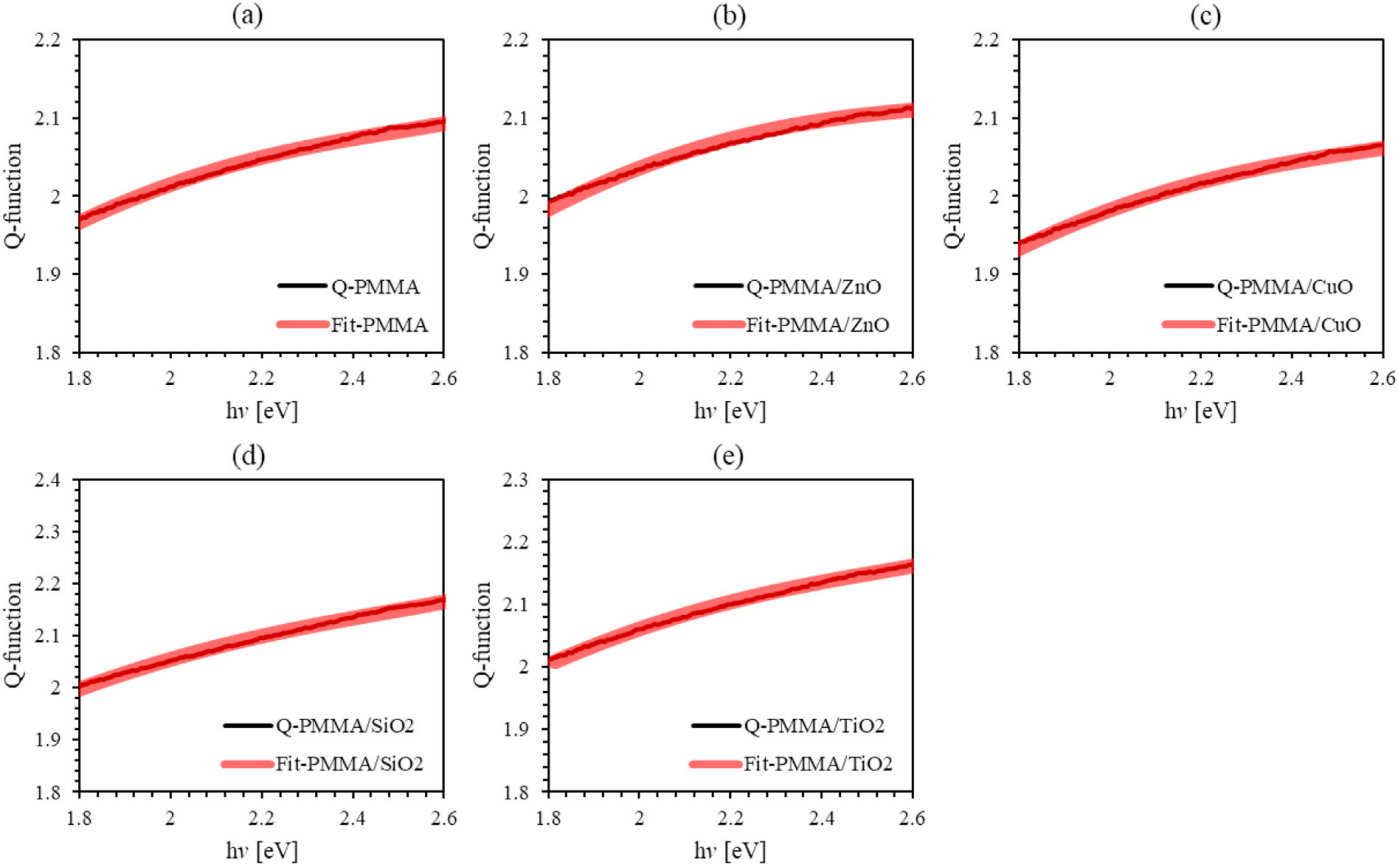
Experimental and fitting of Q-function of PMMA and PMMA incorporated with ZnO, CuO, SiO_2_ and TiO_2_ NPs nanocomposites thin films as a function of incident photon energy: (a) un-doped PMMA, (b) PMMA/ZnO nanocomposite thin films, (c) PMMA/CuO nanocomposite thin films, (d) PMMA/SiO_2_ nanocomposite thin films, (e) PMMA/TiO_2_ nanocomposite thin films. Experimental Q-function is black solid line, and fitting Q-function is red circles.

Q-function of PMMA and PMMA doped with ZnO, CuO, TiO_2_ and SiO_2_ NPs, as well as, our model fit over the low incident photon energy is plotted against the energy of incident photon as shown in Figure 6. The key fitting constants of the proposed mathematical model are given in Table 2. The value of the optical bandgap of PMMA thin film is found to be 4.273 eV in good agreement with that determined using Tauc method [49]. Furthermore, we found that the bandgap decreases as MO NPs are introduced into PMMA matrix as could be seen from Table 2. This is a strong evidence for the ability of the nanocomposites to enhance the ion transfer between MO NPs and PMMA matrix. The values of the bandgap energy of PMMA and the ZnO, CuO, TiO_2_ and SiO_2_ NPs nanocomposites incorporated with PMMA thin films are tabulated in Table 2. The discrepancy between the obtained results and those deduced from classical models could be attributed to the difference between the average thickness calculated and that measured more accurately using UV-vis spectrophotometer.

**Table 2:** The key fitting constants of the mathematical model, Urbach energy and positions of conduction and valence bands of PMMA and PMMA incorporated with ZnO, CuO, SiO_2_ and TiO_2_ NPs nanocomposites thin films.

Parameter	PMMA	PMMA-ZnO	PMMA-CuO	PMMA-SiO2	PMMA-TiO2
A	1.092	1.884	2.378	2.047	1.979
B	67.114	71.384	93.475	78.672	75.384
C	223.247	242.257	315.068	259.801	251.370
Film thickness d(nm)	507.508	492.469	500.635	503.926	505.284
Bandgap energy $E_{g}$(eV) from Q-function	4.273	4.036	3.988	3.972	4.027
Bandgap energy $E_{g}$(eV) from Tauc plot	4.131	4.001	4.000	4.020	4.022
Urbach energy ($E_{U}$) (meV)	164	183	172	143	175
X (PMMA) (eV)	6.504	6.504	6.504	6.504	6.504
X (MONPs) (eV)	--	4.697	6.473	5.812	5.810
X (tot) (eV)	6.504	6.324	6.501	6.435	6.435
E_CB_ (eV)	-0.114	-0.195	0.007	-0.051	-0.102
E_VB_ (eV)	4.123	3.842	3.995	3.921	3.971

#### 4.3.2 Experimental band structure

We determine Urbach energy, $E_{U}$ using Urbach empirical rule [80]. Near optical band edge, $\alpha =\alpha_{0}\;\exp\left(hv/E_{U}\right)$, where $\alpha_{0}$ is a constant, hv is the incident photon energy and $E_{U}$ is Urbach energy [81, 82]. The estimated values of $E_{U}$ are listed in Table 2. For un-doped PMMA thin film $E_{U}$ = 164meV. Its values surges as MO NPs are inserted into PMMA polymeric matrix except for PMMA/SiO_2_ thin film for which $E_{U}$ decreases to 143meV. The increased values of $E_{U}$ indicates a highly disordered thin film [83].

**Figure 7:**
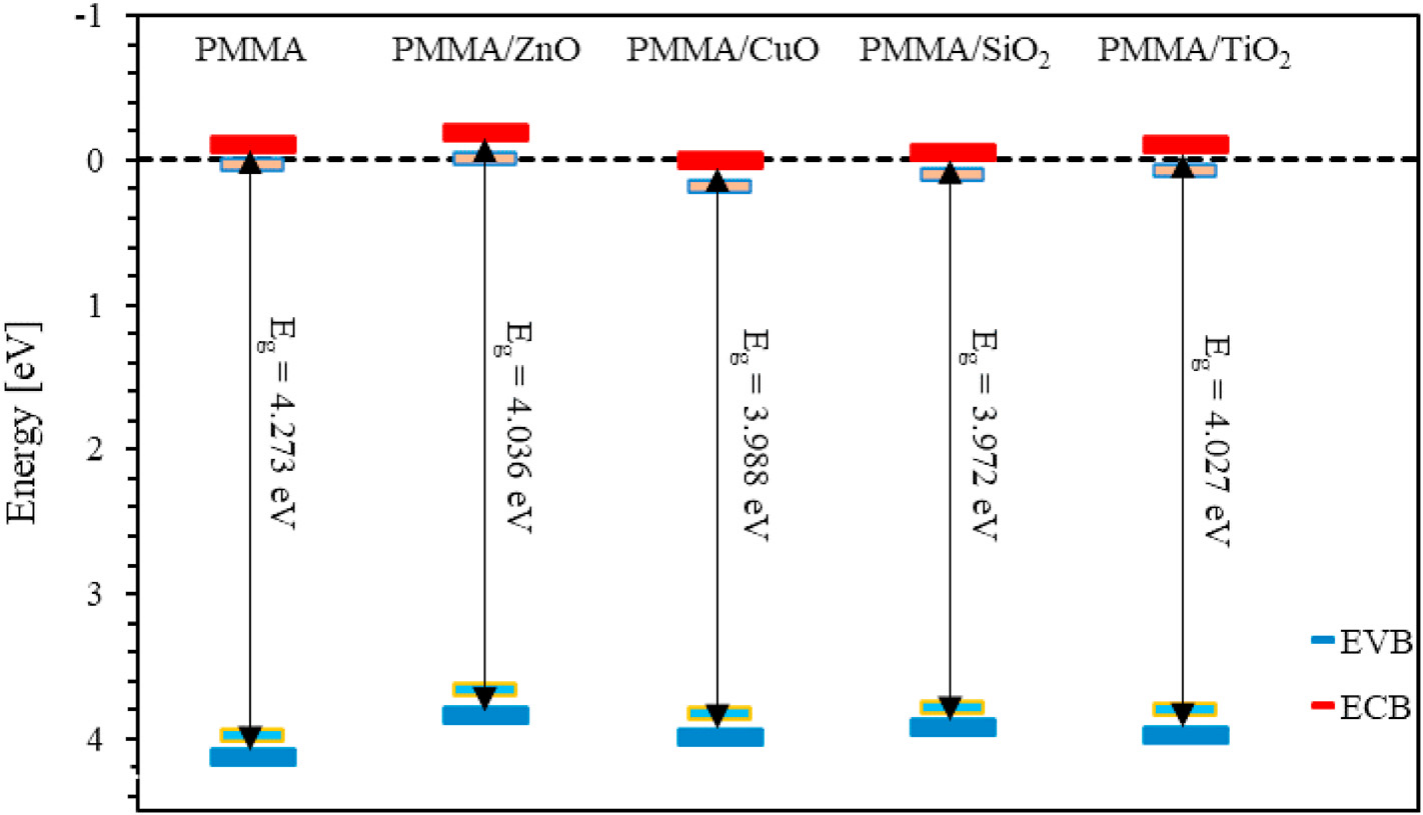
Schematic diagrams of band structures of PMMA and PMMA incorporated with ZnO, CuO, SiO_2_ and TiO_2_ NPs nanocomposites thin films.

For PMMA and ZnO, CuO, SiO_2_ and TiO_2_ NPs nanocomposites incorporated with PMMA thin films, these positions can be evaluated using Eqs. (16), (17), and (18) [84, 85],

(16) $$E_{CB}=X-E_{e}-0.5E_{g}$$

where:

(17) $$X={\left[\left(X_{Z1}^{x_{1}}\right)*\left(X_{Z2}^{x_{2}}\right)*\ldots \right]}^{\left(1/(x_{1}+x_{2}+\ldots \right)}$$

and

(18) $$X_{Z}=\left(\frac{1}{2}\right)\left(E_{EA}^{Z}+E_{Ion}^{Z}\right)$$

where $E_{CB}$ is the conduction band energy, $E_{e}$ is the energy of free electrons of hydrogen scale which equals 4.5eV [84]. The parameter X is an energy parameter that depends on the elements making the nanocomposite thin film, and x is a number of atoms in the molecules. The parameter $E_{g}$ represents the optical bandgap of nanocomposite thin film samples. $E_{EA}^{Z}$ is the electron affinity energy and $E_{Ion}^{Z}$ is the ionization energy of Z element. The calculated values of the energy parameter (X) and the positions of both the conduction and the valence bands of PMMA and the ZnO, CuO, SiO_2_ and TiO_2_ NPs nanocomposites incorporated with PMMA thin films are reported in Table 2 and sketched on an energy diagram as shown in Figure 7. Careful inspection of Figure 7 indicates that the positions of the conduction band minimum (CBM) of PMMA/ZnO, PMMA/CuO, PMMA/SiO_2_ and PMMA/TiO_2_ nanocomposite thin films are located at -0.195 eV, 0.007 eV, -0.051 eV and -0.102 eV, respectively. Therefore, CBMs of these thin films are shifted toward lower negative energy regions with respect to the CBM of the PMMA thin films located at -0.119 eV except for PMMA/ZnO case. Furthermore, the valence band maximum (VBMs) of PMMA/ZnO, PMMA/CuO, PMMA/SiO_2_ and PMMA/TiO_2_ nanocomposite thin films are located at 3.842 eV, 3.995 eV, 3.921 eV, and 3.971 eV, respectively. The VBMs are shifted toward less positive energy regions of the valence band with respect to the VBM of the PMMA thin film located at 4.123 eV. As a result, optical band gap energy of PMMA are largely tuned and engineered by merely controlling the type of the incorporated MO NPs. Figure 7 also illustrates the defect bands formed as an intermediate state in the bandgap of the material. These defects spread from the lower end of the conduction band deeply into the bandgap.

## 5 FTIR spectroscopy

**Figure 8:**
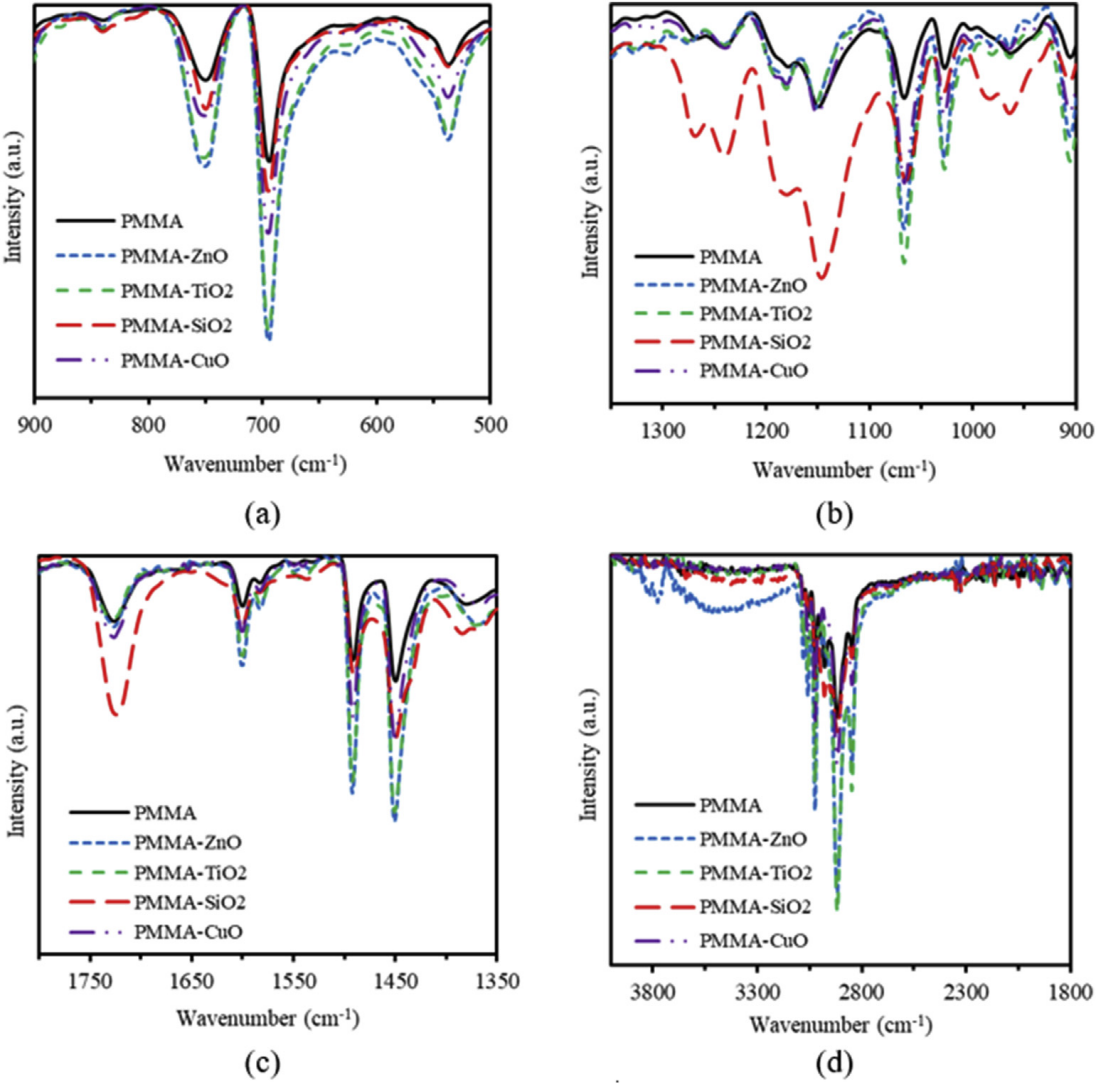
The FTIR spectra pure PMMA, and PMMA incorporated with ZnO, TiO_2_, SiO_2_ and CuO NPs nanocomposites: (a) 500-900 cm^−1^, (b) 900-1350 cm^−1^, (c) 1350-1800 cm^−1^ and (d) 1800-3500 cm^−1^.

Fourier Transform Infrared spectroscopy (FTIR) measurements are performed to investigate the vibrational bands of the nanocomposites. Figure 8 shows the FTIR spectra for PMMA, and ZnO, TiO_2_, SiO_2_ and CuO NPs nanocomposites incorporated in PMMA thin films in the spectral ranges: (a) 500-900 cm^−1^, (b) 900-1350 cm^−1^, (c) 1350-1800 cm^−1^ and (d) 1800-3600 cm^−1^. It is clear that the spectrum in Figure 8(a) exhibits vibrational bands typical for PMMA [86, 87]. Figure 8(a) shows that the peaks observed between 500 cm^−1^ and 800 cm^−1^ could be assigned for C–H bending. Figure 8(b) shows that the peaks observed at 1064 cm^−1^ may correspond to -C-O-C-, while peaks observed between 1181.33 cm^−1^ and 1288.66 cm^−1^ are ascribed to C–O bond stretching vibrations. Figure 8(c) shows that the peaks observed at 1725 cm^−1^ and 1599 cm^−1^ correspond to stretching of C=O and C=C groups, respectively, and the other bands appearing in 1492–1275 cm^−1^ spectral range are associated with CH_3_ and CH_2_ vibrational modes. Finally, the bands that appear in the 3100–2800 cm^−1^ spectral range correspond also to different CH_3_ and CH_2_ vibrational modes as shown in Figure 8(d). The intensity of the peaks at 696 cm^−1^, 747 cm^−1^, 1064 cm^−1^, and that of the peaks between 1350-3600 cm^−1^ increases upon the incorporation of MO NPs into polymeric matrix. The considerable increase in the peak's intensities of the whole FTIR spectra could be attributed to the intermolecular bonding between PMMA matrix and MO NPs. The intensity of the peak at 540 cm^−1^ increases, with a peak position shifted by introducing ZnO, TiO_2_ and CuO NPs can be explained in terms of the existence of Z-O, Ti–O and Cu–O bonds. The elevated intensities of the peaks in the range of 1100–1350 cm^−1^ may be attributed to the existence of Si–O bonds.

## 6 Thermogravimetric analysis (TGA)

**Figure 9:**
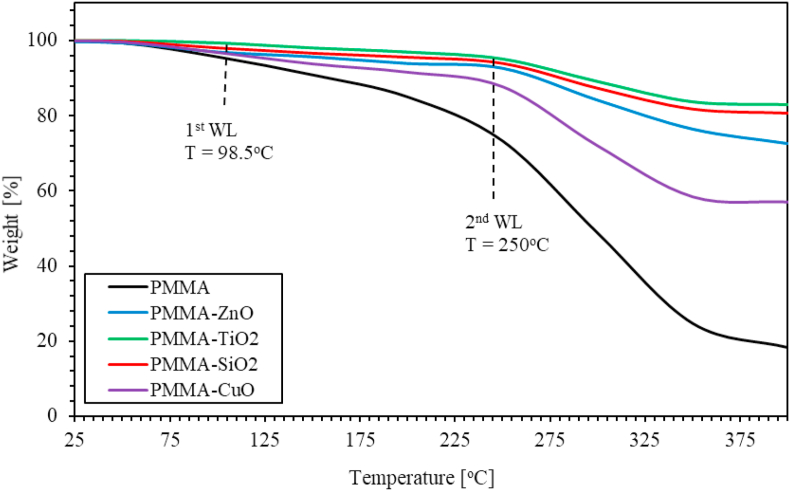
The TGA thermograms of pure PMMA, and PMMA incorporated with ZnO, TiO_2_, SiO_2_ and CuO NPs nanocomposites.

Pure PMMA, and ZnO, TiO_2_, SiO_2_ and CuO NPs incorporated with PMMA thermal stability was investigated by employing thermogravimetric analysis (TGA) at temperatures up to 400 °C. Figure 9 shows TGA thermograms for pure PMMA and for ZnO, CuO, TiO_2_ and SiO_2_ NPs incorporated with PMMA thin films. Two steps of weight loss (WL) at two different temperatures are readily revealed. The first step corresponding to the adsorbed water, while the second step corresponds to intermolecular/intramolecular bonding and chemical stability. PMMA has two weight-loss steps at 98.5 °C and 250 °C. The first WL step was found to be around 20% caused by the adsorbed water molecules and the remaining organic solvent. The second WL step was found to be around 82% related to the decomposed temperature of PMMA. The weight loss decreases from 82% to 43% when CuO NPs are introduced in PMMA matrix, while the weight loss decreased to 27.5% when ZnO NPs are added into PMMA matrix. Moreover, for the PMMA incorporated with TiO_2_ and SiO_2_ NPs, the weight loss decreases from 82% to 17% and 19%, respectively. The weight loss of the nanocomposites decreases as MO NPs are introduced in PMMA matrix could be explained in terms of the strengthening of the physicochemical bonding density in the polymer matrix. It represents the chemical bonding density (i.e., number of bonds, such as covalent and the weaker interactions involving electron-sharing among atoms of the PMMA/MO NPs nanocomposite per unit volume), as well as, the physical binding density that characterizes the number of non-electronic-sharing interactions between atoms such as ionic-based interactions per unit volume.

## 7 Summary and conclusion

In summary, we synthesize PMMA, and ZnO, CuO, SiO_2_ and TiO_2_ NPs nanocomposites incorporated with PMMA thin films with a weight concentration of 10%. SEM images of MO NPs show that all MO NPs have nearly an average size of 50 nm. The optical properties such as optical constants (*n* and *k*), optoelectronic, dispersion, bandgap energy, and the band structure properties of the prepared nanocomposite thin films were determined by analyzing the transmittance and reflectance spectra. The transmittance of PMMA thin film was found to be around 92% in the visible region. We found that, as MO NPs are introduced into PMMA thin films, the transmittance of thin films significantly decreases. Thus, relevant optical parameters are strongly influenced by this decrease. Calculated refractive indices (*n*) of PMMA thin film lie in the range (1.53–1.97) that increase as MO NPs are introduced into PMMA matrices. The major finding of this work is the derivation of a new mathematical model that enables accurate calculation of the optical bandgap energy *E*_g_ and the film thickness utilizing a new defined Q-function that is dependent on the experimental transmittance and reflectance data and is a functional of the photon energy (Q(*E*)). The value of *E*_*g*_ for PMMA thin films is found to be 4.273 eV. As MO NPs are introduced into PMMA thin films, *E*_g_ decreases. These values are in excellent agreement with the values of bandgap determined using Tauc method. The main advantage of the simple mathematical model proposed in this work is the simultaneous accurate determination of the optical bandgap and film thickness. In addition, Urbach energy of PMMA thin film is calculated to be 164meV that increases upon introducing MO NPs into PMMA thin films. However, the PMMA/SiO_2_ thin film exhibits a reduced Urbach energy of 143meV. The increased Urbach energy of MO NPs/PMMA thin films reveals highly disordered thin films. FTIR was used to study the vibrational bands of the nanocomposites and intermolecular bonding between PMMA matrix and MOs NPs. Thermal stability was investigated by employing thermogravimetric analysis (TGA) at temperatures up to 400 °C. Introducing MO NPs into PMMA matrix enhances the thermal stability of the thin films and improves their electronic and optical properties. This evidently contributes positively to the technology of the fabrication of new generation of optoelectronic devices and thin-film transistors.

## Declarations

### Author contribution statement

Qais M. Al-Bataineh, A. M. Alsaad: Conceived and designed the experiments; Performed the experiments; Analyzed and interpreted the data; Contributed reagents, materials, analysis tools or data; Wrote the paper.

Ahmad. A. Ahmad: Conceived and designed the experiments; Performed the experiments; Analyzed and interpreted the data; Contributed reagents, materials, analysis tools or data.

Ahmad D. Telfah: Conceived and designed the experiments; Performed the experiments; Analyzed and interpreted the data.

### Funding statement

This work was supported by 10.13039/501100004035Jordan University of Science and Technology (282-2019).

### Data availability statement

Data will be made available on request.

### Declaration of interests statement

The authors declare no conflict of interest.

### Additional information

No additional information is available for this paper.
